# Cardiovascular benefits of sodium-glucose cotransporter 2 inhibitors in diabetic and nondiabetic patients

**DOI:** 10.1186/s12933-021-01266-x

**Published:** 2021-04-07

**Authors:** Boyang Xiang, Xiaoya Zhao, Xiang Zhou

**Affiliations:** Department of Cardiology, The Second Affiliated Hospital of Soochow University, No. 1055 Sanxiang Road, Suzhou, 215004 People’s Republic of China

**Keywords:** Sodium-glucose cotransporter 2 inhibitors, Cardiovascular benefits, Diabetes

## Abstract

Sodium-glucose cotransporter 2 inhibitors (SGLT2i) were developed as antidiabetic agents, but accumulating evidence has shown their beneficial effects on the cardiovascular system. Analyses of the EMPA-REG OUTCOME trial (Empagliflozin Cardiovascular Outcome Event Trial in Type 2 Diabetes Mellitus Patients) suggested that these benefits are independent of glycemic control. Several large-scale outcome trials of SGLT2i also showed cardiovascular benefits in nondiabetic patients, strengthening this perspective. Extensive animal and clinical studies have likewise shown that mechanisms other than the antihyperglycemic effect underlie the cardiovascular benefits. Recent clinical guidelines recommend the use of SGLT2i in patients with type 2 diabetes mellitus and cardiovascular diseases because of the proven cardiovascular protective effects. Since the cardiovascular benefits are independent of glycemic control, the therapeutic spectrum of SGLT2i will likely be extended to nondiabetic patients.

## Introduction

Sodium-glucose cotransporter 2 inhibitors (SGLT2i) are antidiabetic drugs that lower blood glucose levels in patients with type 2 diabetes mellitus (T2DM). SGLT2i decrease renal glucose reabsorption by blocking SGLT2 and thus enhance the urinary excretion of glucose [1]. Their mechanism of action differs from those of traditional antihyperglycemic interventions, which attenuate insulin sensitivity, preserve β-cell function, or increase tissue glucose uptake.

The concept of SGLTs was first proposed in the 1960s [2] and, during the ensuing decades, six SGLT subtypes, of which SGLT1 and SGLT2 are the most important, were discovered in the human body. SGLT1, which has high affinity and low transport capacity for glucose, is present in the intestine, kidney, heart, prostate, trachea, brain, and skeletal muscle whereas SGLT2, which has low affinity and high transport capacity for glucose, is located almost exclusively in the epithelium of the proximal tubular segment [3–5]. In healthy individuals, almost all filtered glucose (approximately 160–180 g per day) undergoes tubular reabsorption [6]; most (> 90%) is reabsorbed by SGLT2 in the proximal tubule and the remainder (< 10%) is reabsorbed by SGLT1 in more distal segments of the proximal tubule [4, 5, 7]. However, because of a compensatory increase in SGLT1-mediated transport [8], or other latent factors, complete pharmacological blockade of SGLT2 only leads to urinary excretion of 50–80 g of glucose per day (i.e., SGLT2i block < 50% of renal glucose reabsorption) in healthy individuals [4] and this level of SGLT2i-mediated glycosuria does not increase even in those with diabetes mellitus [9, 10].

In addition to improving glycemic control, a growing body of clinical evidence has shown that SGLT2i provide remarkable cardiovascular benefits, most notably a reduced risk of hospitalization for heart failure (HF). These benefits occur rapidly and persist throughout treatment, which is inconsistent with the slow effect of glycemic control on the cardiovascular system [11, 12]. In the EMPA-REG OUTCOME trial (Empagliflozin Cardiovascular Outcome Event Trial in Type 2 Diabetes Mellitus Patients), which included individuals with T2DM and cardiovascular diseases (CVD), only a modest correlation was observed between changes in HbA1c (glycosylated hemoglobin) and amelioration of cardiovascular outcomes, suggesting that the cardiovascular benefits of empagliflozin might be independent of its antihyperglycemic effect [13, 14]. Recently, several large-scale randomized controlled trials of SGLT2i have likewise demonstrated cardiorenal benefits in nondiabetic patients [15–17].

In this review, we focus mainly on the cardiovascular benefits of SGLT2i, the underlying mechanisms, and prospects for clinical application.

## Cardiovascular benefits of SGLT2i

Currently, four SGLT2i (empagliflozin, dapagliflozin, canagliflozin, and ertugliflozin) are licensed by the European Medicines Agency (EMA) and the US Food and Drug Administration (FDA). Several other SGLT2i (e.g., sotagliflozin, remogliflozin, ipragliflozin, and tofogliflozin) have progressed to marketing approval in different regions. Table 1 lists nine large-scale cardiorenal outcome trials of SGLT2i published in the last five years, showing the baseline characteristics of individuals enrolled, interventions, and cardiovascular outcomes.

**Table 1:** Large-scale randomized controlled trials of SGLT2i

Trial name	Population(n)	CVD (%)	T2DM (%)	eGFR	Intervention	Median follow–up (years)	Primary outcomes HR (95% CI)	HHF HR (95% CI)	DCC HR (95%CI)	DAC HR (95%CI)	FNMI HR (95%CI)	FNS HR (95%CI)	Other outcomes HR/AD (95%CI)
EMPA-REG OUTCOME	7,020 with T2DM and established CVD	> 99	100	≥ 30	Empagliflozin 10 mg or 25 mg versus placebo	3.1	MACE 0.86 (0.74–0.99)	0.65 (0.50–0.85)	0.62 (0.49–0.77)	0.68 (0.57–0.82)	0.87 (0.70–1.09)	1.18 (0.89–1.56)	
EMPEROR-Reduced	3,730 patients with HFrEF and NYHA class II–IV symptoms	100	50	NA	Empagliflozin 10 mg versus placebo	1.3	HHF or DCC 0.75 (0.65–0.86)	0.69 (0.59–0.81)	0.92 (0.75–1.12)	0.92 (0.77–1.10)	NA	NA	Change in KCCQ score 1.7 (0.5–3.0)
CANVAS Program	10,142 patients with T2DM and established CVD or multiple cardiovascular risk factors	66	100	≥ 30	Canagliflozin 100 mg or 300 mg versus placebo	2.4	MACE 0.86 (0.75–0.97)	0.67 (0.52–0.87)	0.87 (0.72–1.06)	0.87 (0.74–1.01)	0.89 (0.73–1.09)	0.87 (0.69–1.09)	
CREDENCE	4,401 patients with T2DM and albuminuric chronic kidney disease	50	100	30–89	Canagliflozin 100 mg versus placebo	2.6	ESKD, a doubling of serum creatinine level, or renal or cardiovascular death 0.70 (0.59–0.82)	0.61 (0.47–0.80)	0.78 (0.61–1.00)	0.83 (0.68–1.02)	NA	NA	
DECLARE-TIMI 58	17,160 patients with T2DM and established ASCVD or multiple risk factors for ASCVD	41	100	≥ 60	Dapagliflozin 10 mg versus placebo	4.2	HHF or DCC 0.83 (0.73–0.95); MACE 0.93 (0.84–1.03)	0.73 (0.61–0.88)	0.98 (0.82–1.17)	0.93 (0.82–1.04)	0.89 (0.77–1.01)	1.01 (0.84–1.21)	
DAPA-HF	4,744 patients with HFrEF and NYHA class II–IV symptoms	100	45	≥ 30	Dapagliflozin 10 mg versus placebo	1.5	Hospitalization or urgent visit with intravenous therapy for HF or DCC 0.74 (0.65–0.85)	0.70 (0.59–0.83)	0.82 (0.69–0.98)	0.83 (0.71–0.97)	NA	NA	Change in KCCQ score 1.18 (1.11–1.26); DCC or HHF 0.75 (0.65–0.85)
DAPA-CKD	4,304 patients with an eGFR of 25–75 ml/min/1.73m^2^ and a urinary albumin-to-creatinine ratio of 200–5000	37	68	25–75	Dapagliflozin 10 mg versus placebo	2.4	ESKD, sustained decline in the eGFR of 50%, or renal or cardiovascular death 0.61 (0.51–0.72)	NA	0.81 (0.58–1.12)	0.69 (0.53–0.88)	NA	NA	DCC or HHF 0.71 (0.55–0.92)
VERTIS-CV	8,246 patients with T2DM and ASCVD	100	100	≥ 30	Ertugliflozin 5 mg or 15 mg versus placebo	3.0	MACE 0.97 (0.85–1.11)	0.70 (0.54–0.90)	0.92 (0.77–1.11)	0.93 (0.80–1.08)	1.04 (0.86–1.26)	1.06 (0.82–1.37)	DCC or HHF 0.88 (0.75–1.03)
SOLOIST-WHF	1,222 patients with T2DM and hospitalization for worsening heart failure	100	100	≥ 30	Sotagliflozin 200 mg or 400 mg versus placebo	0.75	Hospitalization or urgent visit for HF or DCC 0.67 (0.52–0.85)	0.64 (0.49–0.83)	0.84 (0.58–1.22)	0.82 (0.59–1.14)	NA	NA	Change in KCCQ score 4.1 (1.3–7.0); Primary outcomes in patients with HFpEF 0.48 (0.27–0.86)

*AD* absolute difference, *ASCVD* atherosclerotic cardiovascular disease, *CI* confidence interval, *CVD* cardiovascular disease, *DAC* death from any cause, *DCC* death from cardiovascular causes, *eGFR* estimated glomerular filtration rate (ml/min/1.73m^2^), *ESKD* end-stage kidney disease, *FNMI* non-fatal or fatal myocardial infarction, *FNS* non-fatal or fatal stroke, *HHF* hospitalization for heart failure, *HF* heart failure, *HFpEF* heart failure with preserved ejection fraction, *HFrEF* heart failure with reduced ejection fraction, *HR* hazard ratio, *KCCQ *Kansas City Cardiomyopathy Questionnaire, *MACE* major adverse cardiovascular events (death from cardiovascular causes, non-fatal myocardial infarction, and non-fatal stroke), *NA* not available, *NYHA* New York Heart Association, *SGLT2* sodium-glucose cotransporter 2, *T2DM* type 2 diabetes mellitus

**Fig. 1:**
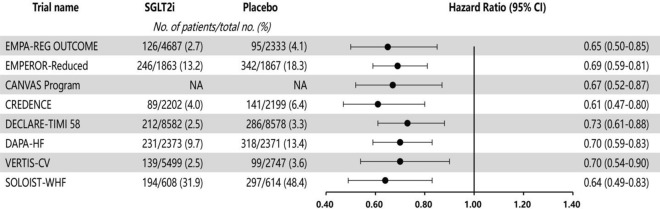
SGLT2i reduce the risk of hospitalization for heart failure in large randomized controlled trials. With SGLT2i therapy, the reduction in risk of hospitalization for heart failure was consistent and significant across different patients. CI, confidence interval; NA, not available

Across the whole patient population (including individuals without T2DM), these randomized controlled trials all demonstrated the cardiovascular benefits of SGLT2i, most notably a remarkable reduction in the risk of hospitalization for HF (Fig. 1), and indicated the involvement of mechanisms other than glycemic control. In addition to improved prognosis, quality of life also improved in patients with HF and reduced ejection fraction (HFrEF), as indicated by improved Kansas City Cardiomyopathy Questionnaire (KCCQ) scores.

### Empagliflozin

The EMPA-REG OUTCOME trial [18] in patients with T2DM and CVD demonstrated that empagliflozin, as compared with placebo, decreased the risk of major adverse cardiovascular events (MACE), an effect that was principally ascribed to a significant reduction in risk of death from cardiovascular causes. Both the risk of hospitalization for HF and death from any cause were also markedly reduced by treatment with empagliflozin. Interestingly, a similar decrease in risk of MACE was observed in the two different dose groups in the trial, probably signifying a small dose–response relationship between the dose of empagliflozin and cardiovascular benefits. However, a marked correlation between changes in HbA1c and drug dose was observed, implying the involvement of mechanisms other than glycemic control in the cardiovascular benefits. This was later confirmed in several analyses of the EMPA-REG OUTCOME trial [13, 14].

The EMPEROR-Reduced trial (Empagliflozin Outcome Trial in Patients with Chronic Heart Failure and a Reduced Ejection Fraction) [15] in patients with HFrEF, together with New York Heart Association class II–IV symptoms, showed that empagliflozin, as compared with placebo, reduced the composite risk of hospitalization for worsening HF or death from cardiovascular causes, mainly driven by the reduced risk of the former, and increased KCCQ score at 52 weeks. The effects were of similar size whether diabetes was present or not, again revealing a cardiovascular benefit beyond the antidiabetic effect. As compared with other similar studies, the EMPEROR-Reduced trial extended the known cardiovascular protection of SGLT2i to patients with more advanced but stable HF.

### Canagliflozin

In the CANVAS (Canagliflozin Cardiovascular Assessment Study) program [19], the risk of MACE in patients with T2DM and CVD or multiple cardiovascular risk factors was reduced by treatment with canagliflozin, albeit without a significant decrease in death from cardiovascular causes or death from any cause. The CREDENCE (Canagliflozin and Renal Events in Diabetes with Established Nephropathy Clinical Evaluation) trial [20], which involved patients with chronic kidney disease (CKD) and T2DM, showed that treatment with canagliflozin improved the composite primary endpoint of end-stage kidney disease, doubling of serum creatinine level, or death from cardiorenal causes. Both trials indicated that canagliflozin markedly decreased the risk of hospitalization for HF. An analysis of the CREDENCE trial also found that the cardiorenal benefits were independent of glycemic control [21].

### Dapagliflozin

The DECLARE-TIMI 58 (Dapagliflozin Effect on Cardiovascular Events-Thrombolysis in Myocardial Infarction 58) trial [22], which involved individuals with T2DM and atherosclerotic CVD (ASCVD) or a high risk of ASCVD, demonstrated a remarkable reduction in risk of hospitalization for HF in the dapagliflozin group compared with the placebo group. The reduction in risk of MACE, death from cardiovascular causes, and death from any cause did not, however, reach statistical significance. In a prespecified study of the DECLARE-TIMI 58 trial, dapagliflozin therapy showed cardiovascular benefits, regardless of levels of biomarkers of myocardial injury and HF (including high sensitivity troponin T and N-terminal pro-brain natriuretic peptide), with more benefits seen in patients with more severe CVD [23]. The DAPA-HF (Dapagliflozin and Prevention of Adverse Outcomes in Heart Failure) trial [16], which involved patients similar to those in the EMPEROR-Reduced trial [15], showed that dapagliflozin provided similar cardiovascular benefits to empagliflozin. The DAPA-CKD (Dapagliflozin and Prevention of Adverse Outcomes in Chronic Kidney Disease) trial [17], which involved patients with an estimated glomerular filtration rate (eGFR) of 25–75 ml/min/1.73 m^2^ and albuminuria, showed that treatment with dapagliflozin led to a reduction in the combined risk of end-stage kidney disease, a decline of ≥ 50% in eGFR, or death from cardiorenal causes, regardless of T2DM. All-cause mortality and the composite risk of death from cardiovascular causes or hospitalization for HF were also reduced.

### Other SGLT2 inhibitors

The VERTIS-CV (Evaluation of Ertugliflozin Efficacy and Safety Cardiovascular Outcomes) trial [24], which recruited patients with T2DM and established ASCVD, showed that ertugliflozin, as compared with placebo, only reduced the risk of hospitalization for HF, without significant reduction in risk of MACE, death from cardiovascular causes, or other cardiovascular outcomes. The reason why the results of the trial did not reach statistical significance remains unclear.

The SOLOIST-WHF (Effect of Sotagliflozin on Cardiovascular Events in Patients with Type 2 Diabetes Post Worsening Heart Failure) trial [25] showed that sotagliflozin, an SGLT2i that also inhibits gastrointestinal SGLT1 to some extent, decreased the risk of death from cardiovascular causes or hospitalization and urgent visits for HF in patients with T2DM and recent hospitalization for worsening HF. Treatment with sotagliflozin also increased the KCCQ score, but the trial was stopped earlier than planned due to loss of funding from the sponsor, which probably limited the statistical power to evaluate some outcomes, such as cardiovascular death.

## Cardiovascular protection mechanisms of SGLT2i

**Fig. 2:**
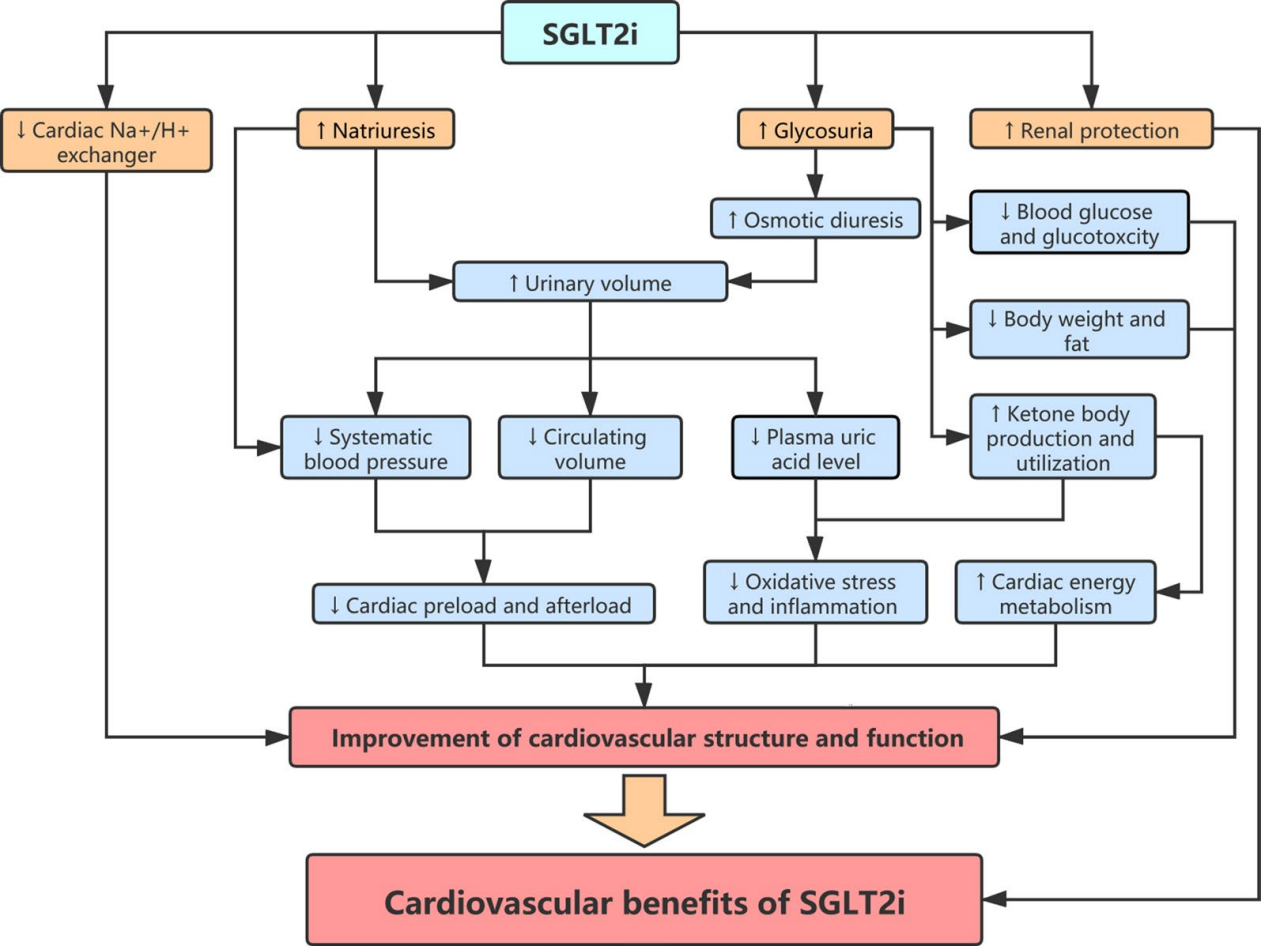
Cardiovascular protection mechanisms of SGLT2i. SGLT2i directly increase natriuresis and glycosuria, leading to a spectrum of secondary beneficial effects on the cardiovascular system. Inhibition of the cardiac Na^+^/H^+^ exchanger and protection of renal function mediated by SGLT2i may also, to some extent, play a beneficial role. These effects jointly contribute to the cardiovascular benefits of SGLT2i, especially the reduced risk of hospitalization for heart failure

The cardiovascular benefits of SGLT2i are mediated by multiple direct and indirect mechanisms that are interwoven and interactional (Fig. 2). These mechanisms improve many aspects of the cardiovascular system, including hemodynamics, metabolism, oxidative stress, and inflammation.

### Glycemic control and attenuation of glucotoxicity

Clinical studies in patients with T2DM have suggested that SGLT2i, as compared with placebo, decrease HbA1c by 0.6–1.0% in the presence of different background therapies [26–28]. Although it remains controversial whether SGLT2i can be combined with insulin to treat type 1 diabetes mellitus (T1DM) because of safety issues, recent meta-analyses of clinical trials have demonstrated a reduction in HbA1c levels of approximately 0.4% in patients with T1DM [29, 30]. The antihyperglycemic effect of SGLT2i depends on urinary glucose excretion and, in patients with CKD, the effect is thus diminished as eGFR decreases [31]. In the CREDENCE trial, the blood glucose lowering effect weakened over time, whereas the cardiovascular benefits did not [20], indicating that glycemic control does not account for the cardiorenal benefits of SGLT2i.

Because urinary glucose levels are elevated, the most common adverse event of SGLT2i is genital infections, with a higher incidence in females than in males [32]. SGLT2i generally do not lead to hypoglycemia [16–19, 22], unless combined with drugs that cause hypoglycemia, such as sulfonylureas [33].

As well as reducing blood glucose, SGLT2i also decrease glucotoxicity, which is manifest as reduced generation of advanced glycation end products (AGEs) and reduced expression of receptors for advanced glycation end products (RAGEs). AGEs are an assortment of heterogeneous compounds that are produced via non-enzymatic glycation and oxidation of proteins, lipids, and nucleic acids [34]. RAGEs are present on the surface of numerous different cardiovascular cells, including smooth muscle cells, endothelial cells, cardiomyocytes, and immune cells, such as monocytes/macrophages and T lymphocytes [35]. Accumulating evidence shows that AGEs mediate detrimental effects on the cardiovascular system by two main mechanisms: crosslinking of tissue proteins and activation of AGE-RAGE signaling pathways [36, 37]. AGEs contribute to vascular stiffness and myocardial fibrosis by crosslinking tissue collagen and elastin [38–40] and enhance oxidative stress and inflammation by binding to RAGEs [41, 42]. Studies in diabetic rodents treated with SGLT2i showed suppression of the AGE-RAGE axis in the kidney [43] and aortic tissues [44] but not in the myocardium [45].

### Natriuresis, diuresis, and reduction in plasma volume

SGLT2i reduce the reabsorption of filtered glucose and sodium by blocking SGLT2, thus leading to natriuresis and osmotic diuresis. The natriuretic effect of SGLT2i may also be enhanced because of functional coordination with Na^+^/H^+^ exchanger 3, which mediates a large fraction of sodium absorption in the proximal tubule [46]. Skin sodium levels, which are closely associated with left ventricular mass [47], are increased in patients with T2DM [48] and SGLT2 inhibition reduced skin sodium levels (without osmotic activity) in T2DM patients [49].

SGLT2i lead to a rapid increase in urine volume (approximately 110–470 ml/day), which settles at a new stable level over 12 weeks [50], with a persistent decline in plasma volume of about 7% by 12 weeks [51]. The gradual attenuation of the diuretic effect is presumably regulated by compensatory mechanisms. A study in rats showed an increase in vasopressin-induced solute-free water reabsorption after administration of SGLT2i [52]. SGLT2i increase the sodium concentration delivered to the macula densa and thus enhance renal tubuloglomerular feedback signals, resulting in a decrease in GFR and contraction of the renal afferent arterioles [53].

SGLT2i attenuate congestion, with little effect on arterial perfusion, in patients with HF. A mathematical model suggested that this is because the osmotic diuresis induced by SGLT2i leads to greater clearance of electrolyte-free water in the intercellular space than in the blood vessels, causing a greater reduction in intercellular fluid volume, relative to circulating volume [54]. The improved quality of life in patients with HF during therapy with SGLT2i might be partly explained by this mechanism.

### Reduction in blood pressure

Recent meta-analyses of clinical research that monitored ambulatory blood pressure show that SGLT2i therapy leads to a decline in systolic and diastolic blood pressure (approximately 3–4 mmHg and 1–2 mmHg, respectively), over study durations of 4–12 weeks. The decrease in blood pressure is greater during the daytime than during the night [55, 56], and the effect does not vanish with more prolonged therapy [15, 16, 57]. The combined effects of SGLT2i on osmotic diuresis and natriuresis are postulated to play a major role in blood pressure lowering [53], although the effect of SGLT2i on the sympathetic nervous system may also contribute to the reduction in blood pressure [58]. Interestingly, the antihypertensive effect remains unchanged regardless of the dose of SGLT2i [55], also indicating little correlation between the dose and the cardiovascular benefits of SGLT2i, as mentioned above. The effect of SGLT2i on blood pressure may also be independent of renal function and glycemic control [59, 60].

Although SGLT2i reduce plasma volume and blood pressure, heart rate is not increased [18, 61, 62], probably implying that SGLT2i decrease the preload and afterload, with cardiac output maintained, or inhibit sympathetic nervous activity.

### Amelioration of endothelial dysfunction and vascular stiffness

Arterial stiffness is strongly associated with hypertension, cardiovascular events, HF, and death [63–65], and endothelial dysfunction plays a vital role in the development of coronary artery disease and HF [66, 67]. Many clinical studies [62, 68–70] have shown that short-term therapy with SGLT2i mitigates aortic stiffness and improves endothelial function. One study [71], however, did not show such benefits, presumably because of differences in design and settings between this and the other studies. A study investigating the effects of long-term treatment is in progress and should further confirm the vascular benefits [72].

### Weight loss and effects on fat

A systematic review of clinical research comparing SGLT2i monotherapy with placebo showed a treatment-related weight loss of approximately 1.5–3 kg [73]. A rapid decrease in body weight was observed during the initial few weeks, followed by a gentle decrease. Weight loss plateaued after 24 weeks and thereafter remained stable [74, 75]. Studies using bioimpedance spectroscopy showed that the weight loss during treatment with SGLT2i could be principally attributed to a decrease in both visceral and subcutaneous adipose tissue [74, 76, 77], with no obvious change in lean tissue mass [78, 79]. This finding is in line with other studies calculating adipose distribution indices [80, 81] or using x-ray absorptiometry [74, 82]. The initial rapid weight loss may be caused by a transient decrease of extracellular water (approximately − 0.5 L of extracellular fluid at 1 month), which gradually normalizes over the next few months [78, 83]. It has been suggested that the reduction in adipose tissue mass after medication with SGLT2i may be caused by an energy loss (around 200–300 kilocalories per day [84, 85]) due to increased glucose excretion [86, 87] and enhanced lipid mobilization [77, 88].

It is noteworthy that the decrease in epicardial adipose tissue mass observed with SGLT2i [89, 90] is independent of the antihyperglycemic effects [91]. In addition to reducing adipose tissue mass, SGLT2i attenuate systemic and adipose inflammation [77]. The accumulation and inflammation of epicardial fat may promote inflammation and fibrosis in the underlying tissues, thereby contributing to atrial tachyarrhythmias, ASCVD, and HF with preserved ejection fraction (HFpEF) [92]. SGLT2i probably generate cardiovascular benefits by blocking these pathogenic mechanisms.

### Protection of renal function

The renal outcome trials of SGLT2i in patients with CKD have shown renal benefits, including preservation of eGFR and a reduction in albuminuria, although a rapid but slight decrease in eGFR was observed during the first month. The renal benefits and cardiovascular benefits were intertwined [17, 20]. Renal dysfunction is related to cardiac remodeling and systolic dysfunction in patients with HFpEF [93]. Cardiac and renal dysfunction are closely linked and partially share pathophysiological mechanisms [94].

### Improvement of cardiac energy metabolism

During the development of HF, the substrate utilized by cardiomyocytes switches from free fatty acids towards glucose. Oxidation of fatty acids produces many adenosine triphosphate (ATP) molecules and has a high demand for oxygen molecules, whereas oxidation of glucose produces less ATP but has higher oxygen efficiency [95, 96]. The switch from free fatty acids to glucose results in an energy deficiency [88], which lowers cardiac work efficiency and worsens HF. In addition to free fatty acids and glucose, β-hydroxybutyrate (a type of ketone body), which is most easily extracted by the myocardium [97], is also utilized by the heart in the fasting state. Oxidation of β-hydroxybutyrate has the highest oxygen efficiency and produces many ATP molecules with the lowest oxygen demand [95, 96]. SGLT2i-induced glycosuria reduces blood glucose and consequently contributes to many metabolic adaptations similar to those in the fasting state, including decreased glucose oxidation, acceleration of lipolysis, augmentation of fat oxidation, and increased plasma concentrations of ketone bodies [88, 98]. SGLT2 inhibition also leads to a myocardial metabolic shift away from glucose toward ketone bodies and free fatty acids. This shift enhances the generation of myocardial energy, thereby improving myocardial remodeling and left ventricular systolic function [99]. The energetic advantage provided by preferential ketone body utilization by cardiomyocytes probably underlies the cardiovascular benefits of SGLT2i, as described in the "thrifty substrate" hypothesis [95].

Clinical studies have shown a slight increase in hematocrit, probably driven by a reduction in plasma volume and a transient elevation in erythropoietin [51]. The increased hematocrit is expected to enhance oxygen delivery to tissues [100] and improve cardiac metabolism. Additionally, ketone bodies, especially β-hydroxybutyrate, can also attenuate systemic inflammation and oxidative stress by inhibiting the Nod-like receptor protein 3 (NLRP3) inflammasome [101, 102] and class I histone deacetylases [103], and by activating G-protein coupled receptor 109 and hydroxycarboxylic acid receptor 2 [104]. Because plasma ketone levels are modestly elevated by SGLT2i [88, 105], the elevation in levels of ketone body exerts salutary effects with little increase in the occurrence of diabetic ketoacidosis [18, 19, 22].

### Inhibition of cardiac Na^+^/H^+^ exchanger

Enhanced cardiac Na^+^/H^+^ exchanger activity is found during the development of HF [106, 107]. Studies in animal models demonstrate that SGLT2i lower cytoplasmic sodium and calcium concentrations and elevate mitochondrial calcium concentration via direct inhibition of the myocardial Na^+^/H^+^ exchanger [108, 109]. Previous animal studies showed that inhibition of the Na^+^/H^+^ exchanger alleviates myocardial hypertrophy and HF [110, 111], and that enhancing mitochondrial calcium concentrations during the development of HF is associated with attenuation of cardiac remodeling and fibrosis and the prevention of sudden cardiac death [112]. Inhibition of the Na^+^/H^+^ exchanger may thus, to some extent, account for the cardiovascular benefits of SGLT2i.

Based on the inhibitory effect of SGLT2i on the Na^+^/H^+^ exchanger, a sodium hypothesis has been put forward. The reduction in mitochondrial calcium concentration, which is secondary to the elevation in intracellular sodium concentration in the failing myocardium, decreases the activity of Krebs cycle dehydrogenases, thus hindering regeneration of the reducing equivalents that plays a key role in matching energy supply to demand. Inhibition of the cardiac Na^+^/H^+^ exchanger by SGLT2i improves the failing myocardium by correcting sodium and calcium handling [113]. The sodium hypothesis is an extension of the "thrifty substrate" hypothesis [95].

### Reduction in serum uric acid level

Uric acid, the end-product of purine metabolism in humans, is largely excreted in urine. SGLT2i-induced glycosuria competitively suppresses uric acid absorption by glucose transporter 9b in the proximal tubule, leading to increased uric acid excretion and reduced plasma levels of uric acid [114, 115]. In a meta-analysis of 62 clinical studies involving 34,391 patients with T2DM, SGLT2i decreased plasma uric acid levels by 15–45 μmol/L. The effect had a rapid onset and persisted during long-term treatment [116]. Increased uric acid stimulates the proliferation and hypertrophy of vascular smooth muscle cells [117], promotes intracellular oxidative stress [118], depletes nitric oxide [119], activates the vascular renin-angiotensin system [120], and induces an inflammatory reaction [121]. Increased uric acid is also associated with hypertension [122], atrial fibrillation [123], and HF [124].

### Improvements in cardiac structure and function

Since there is no expression of SGLT2 in the human heart [125], the underlying effect of SGLT2i on cardiac structure and function is probably mediated largely by hemodynamic, metabolic, and neurohormonal effects. In diabetic mice, SGLT2i reduces expression of pro-fibrotic proteins, decreases deposition of collagen I/III and α-smooth muscle actin in the myocardial interstitium, and improves cardiomyocyte mitochondrial ultrastructure, thereby reducing cardiac fibrosis and hypertrophy, and improving diastolic function [45, 126]. In rats with myocardial infarction, SGLT2i attenuate myocardial fibrosis by activating the signal transducer and activator of transcription 3 (STAT3) pathway and reducing the release of superoxide and nitrotyrosine [127]. In a nondiabetic pig model of HF, SGLT2 inhibition appears to improve cardiac remodeling at the three levels of anatomy, metabolism, and neurohormones, thereby enhancing cardiac systolic function [99].

Many clinical studies using cardiac magnetic resonance imaging or echocardiography have also shown that SGLT2i improve cardiac diastolic function and reduce left ventricular mass and volume [128–131]. However, most studies excluded individuals with overt HF and it, therefore, remains uncertain whether SGLT2i can attenuate advanced left ventricular remodeling.

### Attenuation of inflammation

Low-grade inflammation is recognized to contribute to the development of atherosclerosis and to be associated with an increased risk of CVD [132, 133]. Many studies have indicated that SGLT2i slightly decrease circulating levels of inflammatory factors, including interleukin-6, high‐sensitivity C‐reactive protein, and tumor necrosis factor-γ and -α, in patients with T2DM [90, 134–137]. SGLT2i also reduce M1 macrophage accumulation and polarize M2 macrophages in fat and liver [77]. The anti-inflammatory effect of SGLT2i is probably mediated by many other factors, such as increased levels of ketone bodies and reduced levels of uric acid [138, 139].

### Other possible mechanisms

Detection of increased luminal sodium concentrations by the macula densa would theoretically lower plasma renin levels by reducing the release of renin by juxtaglomerular cells, leading to inhibition of the renin–angiotensin–aldosterone system (RAAS). Animal studies showed that SGLT2i suppressed renal RAAS [140, 141] whereas a clinical study in outpatients with T2DM suggested that SGLT2i transiently enhanced plasma renin activity, which then returned to baseline after 3 months [78]. The effect of SGLT2i on the RAAS thus remains unclear.

A shift in cell life programs from defense to dormancy has been hypothesized to underlie the cardiovascular benefits of SGLT2i. The beneficial effects on the cardiovascular system are suggested to involve aspects of metabolism, hormones, and inflammation [142, 143] but this mechanism is not totally consistent with the "thrifty substrate" hypothesis.

## Prospects for use of SGLT2i in CVD

Extensive clinical studies of SGLT2i consistently suggested an improvement in the quality of life and prognosis of individuals with HFrEF, including more advanced but stable HFrEF. A comparative analysis of three large randomized controlled trials also supports the combined use of an SGLT2i, a mineralocorticoid receptor antagonist, an angiotensin receptor-neprilysin inhibitor, and a β-blocker as a new treatment standard for HFrEF. This new therapeutic regimen produced greater treatment effects than traditional therapy with an angiotensin-converting enzyme inhibitor or angiotensin receptor blocker and β-blocker [144]. In the future, SGLT2i have great potential to be a preferred class of cardiovascular drugs to treat HFrEF.

Almost half of patients with HF have HFpEF [145]. In observational studies, patients with HFpEF have a similar incidence of hospitalization and death to patients with HFrEF, but have better outcomes [146]. Several hemodynamic and molecular mechanisms, such as left atrial hypertension, increased circulating volume, microvascular inflammation, cardiometabolic dysfunction, and cardiac fibrosis, have been suggested to offer potential treatment opportunities for HFpEF [147]. As discussed above, many experimental and clinical studies have shown that SGLT2i have a beneficial role in these aspects of disease, though the subjects in most studies did not have HFpEF. SGLT2i improved left ventricular remodeling and diastolic function in animal models with HFpEF and in cardiac tissues from patients with HFpEF [148, 149]. The SOLOIST-WHF trial demonstrated that sotagliflozin significantly reduced the composite risk of hospitalization or urgent visit for HF or death from cardiovascular causes in patients with HFpEF [25]. Whether other SGLT2i likewise have cardiovascular benefits in patients with HFpEF remains unknown.

The DECLARE-TIMI 58 trial and the VERTIS-CV trial did not show significant improvement in cardiovascular outcomes (except for hospitalization for HF) in patients with established ASCVD or risk factors for ASCVD [22, 24]. Other large clinical studies of SGLT2i also showed no marked reduction in the occurrence of ASCVD, including myocardial infarction and stroke. Consequently, SGLT2i may only have a modest benefit in the treatment of ASCVD.

SGLT2i share many pharmacological advantages, including rapid oral absorption, long half-life, absence of clinically relevant drug–drug interactions, extensive hepatic biotransformation, and low renal clearance of the parent drug [150]. Practice guidelines have recommended using SGLT2i in patients with T2DM and CVD or CKD because of the proven cardiorenal benefits [151, 152].

**Fig. 3:**
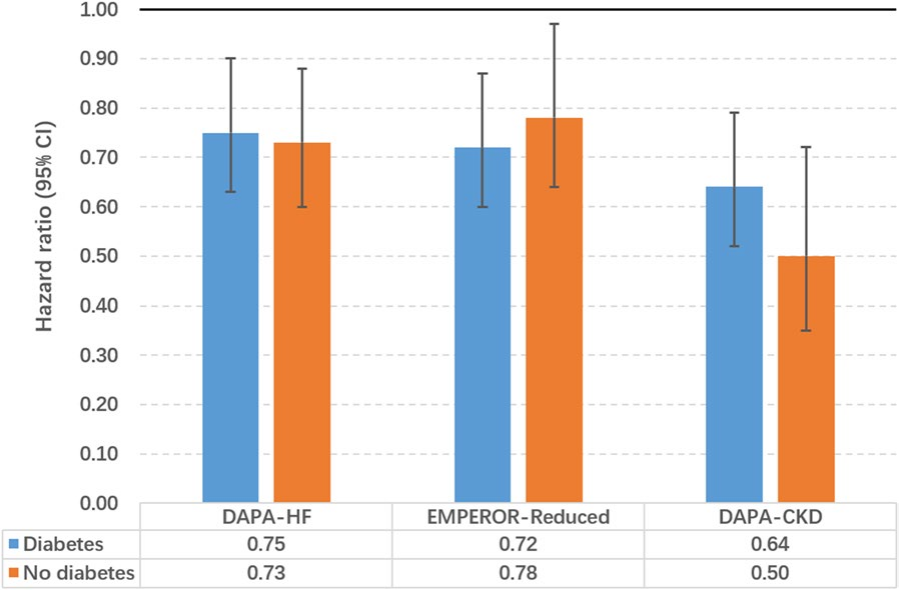
Primary cardiorenal outcomes in diabetic and nondiabetic patients in three large-scale clinical trials. The primary outcome of the DAPA-HF trial is a composite of hospitalization or urgent visit with intravenous therapy for heart failure or death from cardiovascular causes. The primary outcome of the EMPEROR-Reduced trial is a composite of hospitalization for heart failure or death from cardiovascular causes. The primary outcome of the DAPA-CKD trial is a composite of end-stage kidney disease, sustained decline of 50% in eGFR, or renal or cardiovascular death. Cardiorenal protection is seen consistently in patients with or without diabetes. CI, confidence interval

Of note, recent large-scale clinical trials have likewise revealed substantial cardiorenal benefits in patients without T2DM (Fig. 3). The EMPEROR-Reduced trial [15] and the DAPA-HF trial [16] both showed a remarkable reduction in risk of hospitalization for HF or death from cardiovascular causes in nondiabetic patients with HFrEF after treatment with SGLT2i. The DAPA-CKD trial [17] demonstrated renal protection in nondiabetic patients with CKD. These results signify that the therapeutic spectrum of SGLT2i will probably be extended to nondiabetic individuals with HFrEF or CKD.

## Conclusions

Large-scale clinical trials of SGLT2i all showed cardiovascular benefits across different patients, most notably a significant decrease in the occurrence of HF. Because of the proven benefits of SGLT2i, which are independent of glycemic control, many international guidelines recommend the use of SGLT2i in diabetic patients with CVD. SGLT2i directly increase natriuresis and glycosuria, leading to a spectrum of downstream effects. These effects jointly underlie the cardiovascular benefits of SGLT2i. Recently, several large-scale trials have discovered similar cardiovascular benefits in nondiabetic patients with HFrEF. Consequently, SGLT2i will be likely to be used to treat nondiabetic patients with HFrEF.

## Data Availability

Not applicable.

## Abbreviations

AGE: Advanced glycation end product
ASCVD: Atherosclerotic cardiovascular diseases
ATP: Adenosine triphosphate
CANVAS: Canagliflozin Cardiovascular Assessment Study
CREDENCE: Canagliflozin and Renal Events in Diabetes with Established Nephropathy Clinical Evaluation
CVD: Cardiovascular diseases
DAPA-CKD: Dapagliflozin and Prevention of Adverse Outcomes in Chronic Kidney Disease
DAPA-HF: Dapagliflozin and Prevention of Adverse Outcomes in Heart Failure; DECLARE-TIMI 58: Dapagliflozin Effect on Cardiovascular Events-Thrombolysis in Myocardial Infarction 58
eGFR: Estimated glomerular filtration rate
EMA: European Medicines Agency
EMPA-REG OUTCOME: Empagliflozin Cardiovascular Outcome Event Trial in Type 2 Diabetes Mellitus Patients
PEROR-Reduced: Empagliflozin Outcome Trial in Patients with Chronic Heart Failure and a Reduced Ejection Fraction
FDA: Food and Drug Administration
HbA1c: Glycosylated hemoglobin
HF: Heart failure
HFpEF: Heart failure with preserved ejection fraction
HFrEF: Heart failure with reduced ejection fraction
KCCQ: Kansas City Cardiomyopathy Questionnaire
MACE: Major adverse cardiovascular events, including death from cardiovascular causes, non-fatal myocardial infarction, and non-fatal stroke
NLRP3: Nod-like receptor protein 3
RAAS: Renin–angiotensin–aldosterone system
RAGE: Receptor for advanced glycation end products
SGLT2i: Sodium-glucose cotransporter 2 inhibitors
SOLOIST-WHF: Effect of Sotagliflozin on Cardiovascular Events in Patients with Type 2 Diabetes Post Worsening Heart Failure
STAT3: Signal transducer and activator of transcription 3
T1DM: Type 1 diabetes mellitus
T2DM: Type 2 diabetes mellitus
VERTIS-CV: Evaluation of Ertugliflozin Efficacy and Safety Cardiovascular Outcomes
